# A Rare Case of BCL2-Positive Multiple Myeloma Complicated with Follicular Lymphoma

**DOI:** 10.1155/2022/3076968

**Published:** 2022-10-03

**Authors:** Yutaka Tsutsumi, Shinich Ito, Mirei Kobayashi, Takanori Teshima

**Affiliations:** ^1^Department of Hematology, Hakodate Municipal Hospital, 1-10-1 Minato-cho, Hakodate 041-8680, Japan; ^2^Department of Hematology, Hokkaido University Graduate School of Medicine, Sapporo, Japan

## Abstract

A 68-year-old woman presented with follicular lymphoma complicated by IgG kappa-positive multiple myeloma. In this case, both follicular lymphoma and plasma cells were positive for BCL2 by immunostaining. T-cell association in the FL and MM was also analyzed in this case. Some CD3-positive T-cells were found around the plasma cells. These cells were mainly CD8-positive T-cells and not CD4-positive T-cells. These results suggest that CD4-positive T-cells were not associated with the proliferation of the plasma cells in this case. Although the FL that developed was initially positive for BCL2 protein, this does not indicate that plasma cells were derived from FL cells because of the eventual complication that occurred in this case. Furthermore, in this case, rituximab and bendamustine were effective for FL. They were not effective for MM, however, demonstrating that additional treatment options are necessary for the simultaneous treatment of BCL2-positive MM with FL.

## 1. Introduction

Reports of multiple myeloma (MM) merged with malignant lymphoma (ML) are rare [1–3], and reports of MM merged with follicular lymphoma (FL) are even rarer [4].

Here, we report a 68-year-old woman with follicular lymphoma complicated by IgG kappa-positive myeloma. It was reported that marginal zone lymphoma (MALT) is differentiated into plasma cells by reactive CD4 T-cells in MALT patients [5, 6].

In the present case, both follicular lymphoma and plasma cells were positive for BCL2 by immunostaining. On the other hand, CD4-positive T-cells were not associated with the proliferation of the plasma cells. Although the MM that developed was initially positive for BCL2 protein, this does not indicate that plasma cells were derived from FL cells because of the eventual complication that occurred in this case. In the treatment, although rituximab and bendamustine were effective for the FL, they had no effect on the MM. Other treatment options may be necessary for the simultaneous treatment of BCL2-positive MM with FL.

## 2. Case Presentation

**Figure 1:**
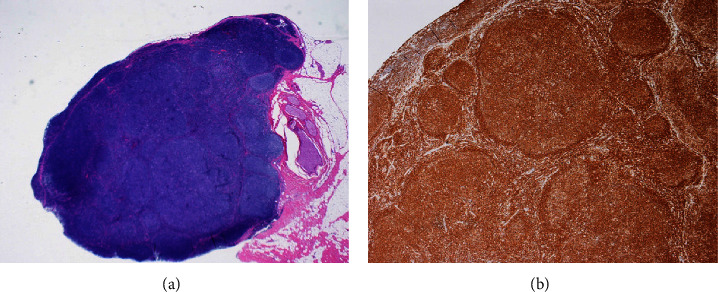
(a) Lymph node specimen from this case; the nodular pattern was observed (×2). (b) Tumor cells were positive for BCL2 (×4).

**Figure 2:**
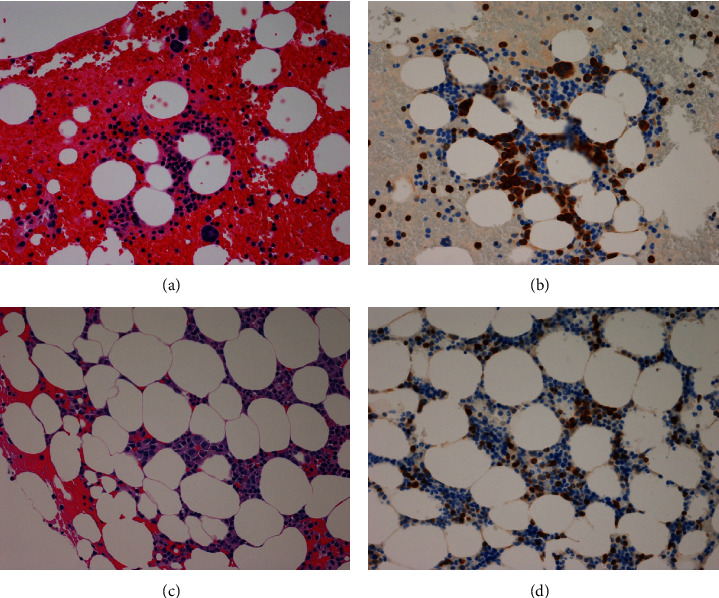
Bone marrow specimens in 2021: (a) atypical plasma cells with lymphocytes (×20). (b) Both lymphocytes and plasma cells are BCL2-positive (×20). Bone marrow specimens in 2016: (c) lymphocytes and plasma cells are observed (×20). (d) Plasma cells. Both cells and lymphocytes are BCL2-positive (×20).

**Figure 3:**
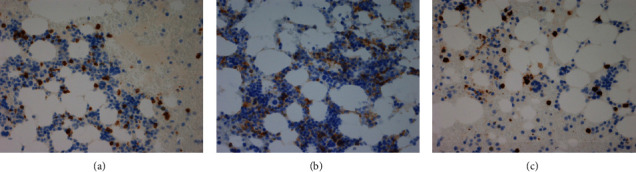
Bone marrow specimens in 2021: (a) CD3-positive T-cells around the myeloma cells (×40). (b) CD4-positive T-cells around the myeloma cells did not increase in number (×40). (c) CD8-positive T-cells around the myeloma cells (×40).

A 68-year-old woman with cervical lymphadenopathy visited our hospital in September 2016. She was diagnosed with follicular lymphoma grade 1 by biopsy of cervical lymphadenopathy (Figures 1(a) and 1(b)). The fusion gene IgH-BCL2 was detected by polymerase chain reaction (PCR) in a bone marrow examination. These suggested the bone marrow invasion by FL and we determined the clinical stage was IV, but the patient was followed closely without chemotherapy due to no symptoms. Plasma cells were 6.4% of this bone marrow specimen. In 2021, an increase in soluble interleukin 2 receptors (sIL2R: 834 U/mL) and *β*2 microglobulin (*β*2MG: 3.54 mg/L) was observed. An enlarging swelling of lymph nodes was also observed by computed tomography (CT). The patient had a white blood cell (WBC) count of 4 × 10^3^/*μ*L, a red blood cell (RBC) count of 3.99 × 10^6^/*μ*L, a hemoglobin (Hb) level of 12.2 g/dl, a platelet count (PLT) of 7.4 × 10^4^/*μ*L, and lactate dehydrogenase (LDH) of 181 U/L at the time of the treatment. A bone marrow examination and immunoelectrophoresis, therefore, were performed for reassessment. Immunoelectrophoresis was positive for immunoglobulin G-kappa (IgG-k), and the presence of neoplastic plasma cells was suspected. A bone marrow examination was performed to confirm IgH-BCL2 positivity. Furthermore, infiltration of B lymphocytes, nuclear enlargement, and 12.2% of multinucleated plasma cells were observed (Figure 2(a)). Serum calcium and creatine albumin levels were within normal limits, the osteolytic lesion was not visible in both CT and positron emission tomography (PET), and the free light chain (FLC) *κ*/*λ* ratio was 28.17 (501/17 mg/L). Although there were more dysplastic plasma cells, these results suggested the FL was complicated with smoldering MM (by IMWG criteria). These results showed that thrombocytopenia was due to FL in this case. Both lymphocytes and plasma cells were found to be BCL2-positive by immunostaining (Figure 2(b)). These observations indicated a follicular lymphoma complication with myeloma. The bone marrow specimen in 2016 was restained, and both lymphocytes and plasma cells were also found to be BCL2-positive (Figures 2(c) and 2(d)). In the bone marrow specimen from 2021, CD3-positive cells were observed around the plasma cells. These T-cells were mostly CD8-positive T-cells, wherein CD4-positive cells did not increase in number (Figures 3(a)–3(c)).

For the present case, rituximab at 375 mg/m^2^ (day 1) and bendamustine at 90 mg/m^2^ (days 2, 3) were administered in four courses for follicular lymphoma. After partial remission, rituximab maintenance therapy was performed once every 2-3 months. However, IgG remained around 3000 mg/dL, the FLC *κ*/*λ* ratio was 24.95 (297/11.9 mg/L), plasma cells were 16.0% after treatment, and no therapeutic effect of rituximab and bendamustine for smoldering MM was observed.

## 3. Discussion

Multiple myeloma is rarely associated with malignant lymphoma, and MALT, AITL, and FL have also been reported as associated with lymphoma [1–4]. Among these lymphomas, it has been reported that MALT is differentiated into plasma cells by reactive CD4 T-cells [5, 6]. In cases where AITL is complicated with MM, AITL itself may promote plasma cell proliferation [7–13]. In these reports [7–13], the proliferation of plasma cells with clonality was observed in five cases, and some involvement of T-cells was suspected, as in the case of MALT mentioned above [5, 6]. On the other hand, only one case of FL has been reported. The reason for the merging with MM is unknown; i.e., it is unknown whether it is derived from FL, and it is unknown whether activated CD4 T-cells were involved [4].

Likewise, in the present case, it was determined that FL was associated with MM from the beginning, but CD4 T-cells were not particularly abundant around plasma cells and B lymphocytes in the analysis of pathological specimens. Therefore, the involvement of CD4 T-cells in plasma cell proliferation and tumorigenesis is unknown.

On the other hand, both follicular lymphoma and plasma cells were positive for BCL2 by immunostaining. Puthier et al. reported high expression of BCL2 in slowly proliferating MM cells [14]. Zeng et al. reported that 43–75% of myeloma cells were BCL2-positive [15]. Thus, these reports show that although the FL that developed was initially positive for the BCL2 protein, the plasma cells were not derived from FL cells in this case.

As previously reported, rituximab and bendamustine were effective for FL. They were not effective for MM, however, demonstrating that MM requires a separate treatment. On basis of the positivity for the BCL2 protein, venetoclax could be one of the treatments options for this case. The effectiveness of venetoclax, however, was limited in the treatment of follicular lymphoma [16, 17]. In a report of FL complicated with MM, rituximab and bendamustine were administered for the treatment of FL, followed by lenalidomide and dexamethasone for the treatment of MM [4]. Although the BCL2 protein was not analyzed, lenalidomide was a useful treatment option for both FL and MM [17].

Further cases need to be examined to clarify treatment options for FL complicated with MM, where both tumor cells are positive for the BCL2 protein.

## Data Availability

The data used to support the findings of this study are included within the article.
